# Cardiac arrhythmias in Dravet syndrome: an observational multicenter study

**DOI:** 10.1002/acn3.51017

**Published:** 2020-03-24

**Authors:** Sharon Shmuely, Rainer Surges, Robert M. Helling, W. Boudewijn Gunning, Eva H. Brilstra, Judith S. Verhoeven, J. Helen Cross, Sanjay M. Sisodiya, Hanno L. Tan, Josemir W. Sander, Roland D. Thijs

**Affiliations:** ^1^ Stichting Epilepsie Instellingen Nederland ‐ SEIN Achterweg 5, 2103 SW Heemstede Dokter Denekampweg 20, 8025 BV Zwolle The Netherlands; ^2^ NIHR University College London Hospitals Biomedical Research Centre UCL Queen Square Institute of Neurology Queen Square London WC1N 3BG UK; ^3^ Department of Epileptology University Hospital Bonn Bonn Germany; ^4^ Centre for Rare Diseases Bonn (ZSEB) University Hospital Bonn Bonn Germany; ^5^ Department of Medical Genetics University Medical Centre Utrecht Heidelberglaan 100 3584 CX Utrecht The Netherlands; ^6^ Academic Centre for Epileptology Kempenhaeghe 5590AB Heeze Heeze The Netherlands; ^7^ UCL NIHR BRC Great Ormond Street Institute of Child Health (ICH) 30 Guilford St London WC1N 1EH UK; ^8^ Chalfont Centre for Epilepsy Bucks SL9 0RJ UK; ^9^ Heart Centre Department of Experimental and Clinical Cardiology Amsterdam University Medical Centres Meibergdreef 9 1105 AZ Amsterdam The Netherlands; ^10^ Netherlands Heart Institute Moreelsepark 1 3511 EP Utrecht The Netherlands; ^11^ Department of Neurology Leiden University Medical Centre Albinusdreef 2 2333 ZA Leiden The Netherlands

## Abstract

**Objectives:**

We ascertained the prevalence of ictal arrhythmias to explain the high rate of sudden unexpected death in epilepsy (SUDEP) in Dravet syndrome (DS).

**Methods:**

We selected cases with clinical DS, ≥6 years, *SCN1A* mutation, and ≥1 seizure/week. Home‐based ECG recordings were performed for 20 days continuously. Cases were matched for age and sex to two epilepsy controls with no DS and ≥1 major motor seizure during video‐EEG. We determined the prevalence of peri‐ictal asystole, bradycardia, QTc changes, and effects of convulsive seizures (CS) on heart rate, heart rate variability (HRV), and PR/QRS. Generalized estimating equations were used to account for multiple seizures within subjects, seizure type, and sleep/wakefulness.

**Results:**

We included 59 cases. Ictal recordings were obtained in 45 cases and compared to 90 controls. We analyzed 547 seizures in DS (300 CS) and 169 in controls (120 CS). No asystole occurred. Postictal bradycardia was more common in controls (*n* = 11, 6.5%) than cases (*n* = 4, 0.7%; *P* = 0.002). Peri‐ictal QTc‐lengthening (≥60ms) occurred more frequently in DS (*n* = 64, 12%) than controls (*n* = 8, 4.7%, *P* = 0.048); pathologically prolonged QTc was rare (once in each group). In DS, interictal HRV was lower compared to controls (RMSSD *P* = 0.029); peri‐ictal values did not differ between the groups. Prolonged QRS/PR was rare and more common in controls (QRS: one vs. none; PR: three vs. one).

**Interpretation:**

We did not identify major arrhythmias in DS which can directly explain high SUDEP rates. Peri‐ictal QTc‐lengthening was, however, more common in DS. This may reflect unstable repolarization and an increased propensity for arrhythmias.

## Introduction

Dravet syndrome (DS; OMIM #607208) is a severe childhood‐onset developmental epileptic encephalopathy, with multiple seizures types. People with DS face a substantial risk of early epilepsy‐related death, of up to 15% by age 20 years.^1^, ^2^ Sudden unexpected death in epilepsy (SUDEP) is common, accounting for nearly half of all deaths^2^ and affecting one in 107 individuals per year.^1^ The underlying pathophysiology of SUDEP is poorly understood, but likely heterogeneous and multifactorial.^3^ It is a seizure‐related event and a high frequency of convulsive seizures (CS) is the major risk factor.^3^

In >70% of people with DS, heterozygous loss‐of‐function mutations are found in the *SCN1A* gene, encoding the α‐subunit of the voltage‐gated sodium channel Nav1.1 in the mammalian brain and heart.^4^, ^5^, ^6^ Other so called “cardiocerebral channelopathies,” are known to have features related to both organs which may lead to sudden death – for example, mutations in *SCN5A* have been linked to epilepsy and long QT syndrome.^7^, ^8^ Mouse models support the strong association between DS and SUDEP.^9^, ^10^, ^11^ Reports of postictal bradycardia^12^, ^13^, ^14^ and ventricular fibrillation^13^ progressing to fatal asystole in DS mice suggest that the mutation not only alters cortical excitability but also increases the propensity to arrhythmias.^12^, ^13^, ^14^ Interictal data in people with DS showed increased QT dispersion and decreased heart rate variability (HRV),^15^, ^16^, ^17^, ^18^ which may increase risk of lethal ventricular dysrhythmias.^19^, ^20^ We prospectively performed long‐term ECG recordings in DS cases and compared them to historical epilepsy controls to find an explanation for the high SUDEP rate. We determined the prevalence of seizure‐induced arrhythmias and repolarization/conduction abnormalities and assessed peri‐ictal HR and HRV.

## Methods

### Subject and seizure selection

We selected individuals with clinical DS and confirmed pathogenic *SCN1A* mutation. Other inclusion criteria were: (1) ≥6 years of age, (2) ≥1 seizure per week averaged over the previous year (all types except absences or myoclonic seizures), and (3) no history of self‐harm. Cases were recruited from participating centers in the Netherlands (SEIN, Heemstede and Zwolle; Kempenhaeghe, Heeze), Germany (University Hospital of Bonn), and the United Kingdom (Great Ormond Street Hospital for Children, London).

All cases had two baseline 12‐lead ECGs (standard and Brugada^21^ lead placement) and wore a 3‐lead ECG device continuously for 20 days (nECG MINDER, Nuubo®, Madrid, Spain; sampling frequency 250Hz and including a tri‐axial accelerometer). The device was attached to a comfortable vest with textile electrodes, allowing for normal daily activities; only during showering the device was not recording. Data were recorded per 24 h (then the device had to be charged and a new recording file was created when the device was turned back on). Carers and participants were asked to record seizure details (e.g., seizure time and type) and the time the person got out and into bed. All continuous data were saved on a microSD card and could be downloaded at the end of the recording period. All ECG data was inspected visually (page by page; each page was a 10‐s window). During inspection the corresponding tachogram was visible below the ECG. Reported seizures were identified based on diary notes, accelerometry, and HR profiles. A HR increase of ≥10% was required to recognize seizures. We additionally included “unreported seizures” with HR patterns resembling a reported CS of that person if: (1) HR changes did not coincide with a sudden body position change (as indicated by the triaxial accelerometer) and (2) seizures in this person were known to be sometimes missed (e.g., found in a postictal state).

Historical controls were selected from a video‐EEG database. A 1‐lead ECG was measured during EEG recording. Two controls were selected for each case, as fewer seizures were expected in controls due to shorter recordings. Inclusion criteria were: (1) ≥6 years of age, (2) definite diagnosis of epilepsy, (3) no suspicion of DS, (4) ≥1 major motor seizure (CS (i.e., focal to bilateral or generalized tonic‐clonic), generalized or focal motor seizures), and (5) postictal recording of >5 min. Controls were frequency matched to mean age and sex ratio of the Dravet group, as these variables may affect cardiac function. Clinical seizures (except absences and myoclonic jerks) which resulted in a HR increase of ≥10% were included.

The following data were obtained from the medical records: sex, age, epilepsy duration, seizure frequency, seizure cluster (yes/no), occurrence (nocturnal/diurnal/both), anti‐seizure medications (ASM), vagal nerve stimulation (yes/no), and etiology. Additionally, for DS, we recorded *SCN1A* mutation type, age of onset developmental delay, and family history of febrile seizures, epilepsy, and sudden cardiac death.

The study protocol was independently approved by the local Medical Ethics Committee of each participating center. A written informed consent was obtained from the participants or assent from parents or legal guardians in case of minors or those with learning disability. The study was registered at ClinicalTrials.gov (NCT02415686).

### Electrocardiographic analysis

Baseline 12‐lead ECG recordings of DS cases were evaluated by one experienced cardiologist (HLT). The ECG of all seizures in cases and controls were assessed manually for abnormalities from 1 min before onset to 5 min after end of seizure (by SS and, in case of uncertainty, by HLT).

All ECG data were imported into MATLAB to enable assessment in the same viewer, with the same measuring tools. HR changes were used to determine the timing of seizure onset and end. We noted peri‐ictal timepoints: **T1** just before seizure onset, **T2** immediately after seizure end, **T3** 2 min, and **T4** 5 min after seizure end. When an ECG abnormality was identified, the cardiologist consulted and if deemed necessary referral for further cardiac evaluation arranged.

#### Analysis part 1: Bradyarrhythmias and QTc‐intervals

The main study parameters were postictal asystole (sinus arrest of ≥3s) and bradycardia (<2nd HR percentile for age; average of three consecutive RR intervals; female 6–8 years 68 bpm, 8–12 years 58 bpm, 12–16 years 54 bpm; male 6–8 years 62 bpm, 8–12 years 55 bpm, 12–16 years 48 bpm; >16 years 50 bpm^21^). Bradycardia and asystole detection were done manually (SS).

We manually measured QT intervals and RR intervals at time points T1–4. QT intervals and RR intervals were averaged from three successive ECG complexes. Four correction formulas (Bazett, Fridericia, Hodges, and Framingham) were used to calculate QTc intervals. All formulas are known to over‐ or undercorrect QT intervals.^23^ To reduce bias error of putatively pathologic intervals, we considered only those on which Bazett and at least one other formula were abnormal. Table 1 shows the QTc parameters that were determined for each seizure. QTc‐lengthening and ‐shortening of ≥60 ms was determined by subtracting QTc at T2–4 from the preictal value (T1). We noted the occurrence of clinically significant prolonged QTc (defined as: ≤13 years ≥ 460 ms, males > 13 years ≥ 470 ms and females > 13 years ≥ 480 ms), shortened QTc (≤340 ms), and marked prolongation (≥500 ms) and shortening (≤300 ms) at every time point.^23^

**Table 1:** Overview of QTc parameters that were determined for each seizure.

QTc parameters	
Marked prolongation	Marked shortening
≥500 ms	≤300 ms
Clinically significant prolonged	Clinically significant shortened
≥460 ms ≤ 13 years	≤340 ms
≥470 ms > 13 years male	
≥480 ms > 13 years female	
Lengthening of ≥ 60 ms	Shortening of ≥ 60 ms
T2 versus T1	T2 versus T1
T3 versus T1	T3 versus T1
T4 versus T1	T4 versus T1

T1, time of seizure onset; T2, seizure end; T3, two minutes after seizure end; T4, five minutes after seizure end.

#### Analysis part 2: Peri‐ictal heart rates, heart rate variability, QRS‐width, and PR‐interval in convulsive seizures

This analysis was only done in CS. For most cases, one of the three leads had good recording quality and could be used for all subsequent analyses. In case of prominent ECG artifacts, all leads were visually inspected to determine the least affected lead (could vary between and within seizures).

Consecutive RR intervals were automatically determined by one of the two peak detection methods. First, the Pan–Tompkins QRS detection algorithm was used and the results were visually inspected.^25^ If there were quality concerns a second detection method based on the Hilbert–Huang transform was applied.^25^ Additional filtering was applied in individual cases depending on signal quality (e.g., removal of low frequencies as only R peaks are needed).

Peri‐ictal HR was determined automatically in all CS at T1–T4 by averaging 10 RR intervals. In case a HR was aberrant, it was checked and measured manually (e.g., all extreme values <50 bpm or >180 bpm or inexplicable values like higher HR at T2 than at T1).

We estimated HRV from 1‐minute windows during three periods: pre‐ictal, postictal, and resting state. We visually inspected the HR segments and selected the most optimal QRS detection method. Segments with insufficient quality of ECG were excluded from further analysis. RR intervals > 3 SD from the average were considered artefacts; these beats and their corresponding interbeat intervals were excluded from analysis. The windows were only analyzed if the summed RR intervals ≥ 50s. The windows were selected as follows:

- The preictal minute was the minute immediately before T1.
- The post‐ictal minute was selected between T3 and T4, depending on the ECG quality. A 1‐minute sliding window was moved along the postictal ECG with 1 s step (starting at T3) until an adequate window was reached (summed RR intervals ≥ 50 s).
- The resting‐state minute required an awake state in a lying position (between 5 and 15 min after onset of supine rest as determined by video inspection (controls) or diary and accelerometry (cases)) and that no seizure had occurred in the previous hour and no CS in the previous 6 h.^27^

The following HRV measures were estimated: average RR interval, root mean square of successive differences of RR intervals (RMSSD), the standard deviation of RR intervals (SDNN), and the percentage of consecutive RR intervals differing by ≥50 ms (pNN50).

QRS‐widths and PR‐intervals were measured manually at T1 and T4. We noted whether these values were pathologically prolonged (>98th percentile for age: QRS‐width female 6–8 years 95 ms, 8–12 years 99 ms, 12–16 years 106 ms; male 6–8 years 98 ms, 8–12 years 103 ms, 12–16 years 111ms; all > 16 years 120 ms; PR‐interval female 6–8 years 156 ms, 8–12 years 163 ms, 12–16 years 176 ms; male 6–8 years 160 ms, 8–12 years 174 ms, 12–16 years 178 ms; all > 16 years 200 ms).^22^

### Statistical analysis

Clinical characteristics were described with means, SDs, medians, (interquartile) ranges, frequencies, and percentages, and compared between groups using the two‐sided unpaired *t*‐test or Mann–Whitney U test for continuous variables and chi‐square for categorical data. To determine whether DS was independently associated with the occurrence of bradycardia, asystole, and QTc variables, we compared these to the controls using generalized estimating equation (GEE) logistic models (analysis part 1). Preictal and postictal HR and HRV variables in DS were compared to controls using GEE linear models (analysis part 2). GEE models were applied to correct for within‐person correlation, seizure onset from sleep or wakefulness, and (in analysis part 1 only) for the presence of CS (yes/no). Resting‐state HRV variables were compared using unpaired *t*‐tests or Mann–Whitney U test. For visual comparison, boxplots were constructed for HRs at T1‐4 of CS for both groups. Normality tests were performed, and logarithmic transformation was applied to right‐skewed‐dependent variables. Outliers were defined as values that lie more than three SD from the mean. The Holm–Bonferroni method was used to correct for multiple comparisons within the different data sets; adjusted p‐values are shown. All tests were two‐tailed and a value of *P* < 0.05 was considered significant. Statistical analyses were performed with IBM SPSS Statistics 24 (IBM Corp. Armonk, NY).

## Results

### Subjects

**Figure 1:**
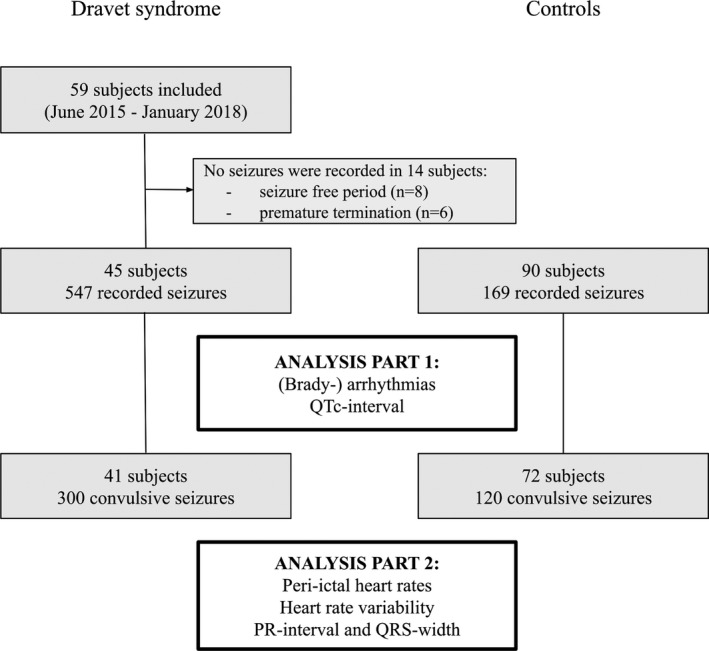
Study flowchart.

Fifty‐nine people were included from June 2015 to January 2018 (48 in the Netherlands, nine in Germany and two in the UK; Fig. 1). No seizures were recorded in eight cases and in six the study was terminated prematurely prior to any seizure thus leaving 45 cases with ictal ECG recordings. Mean age was 19 years (±10 years) and 23 were female (51%; Table 2). In 90 controls, the mean age was 20 years (±9 years) and 46 were female (51%). Most controls were refractory to treatment (*n* = 82, 91%) and 25 controls had a learning disability (28%). A total of 22 different ASM were used between the groups and most were using more than one ASM (DS *n* = 41 (91%) and controls *n* = 57 (63%)). Table s1 shows the *SCN1A* variants.

**Table 2:** Clinical characteristics of Dravet syndrome cases and historical epilepsy controls.

Characteristics	Dravet syndrome (*n* = 45)	Controls (*n* = 90)	*P*‐value
Sex, *n* (%) female	23 (51)	46 (51)	1
Age, years mean (SD)	19 (10)	20 (9.4)	0.54
Epilepsy duration, years mean (SD)	19 (12)	9 (6.5)	<0.001
Seizure frequency per month, median (IQR)	12 (8–25)	8 (3–30)	0.09
Seizures predominantly, *n* (%)^1^			0.001
Nocturnal	26 (58)	26 (29)	
Diurnal	4 (9)	31 (34)	
Both	15 (33)	33 (37)	
Number of ASM, median (range)	3 (0–4)	2 (0–4)	<0.001
ASM type			
Valproic acid	35 (78)	27 (30)	<0.001
Clobazam	28 (62)	19 (21)	<0.001
Stiripentol	19 (42)	1 (1)	<0.001
Topiramate	14 (31)	3 (3)	<0.001
Lamotrigine	3 (7)	28 (31)	0.001
Carbamazepine	2 (4)	24 (27)	0.002
Oxcarbazepine	2 (4)	21 (23)	0.006
Lacosamide	0	8 (9)	0.039
Drug tapering, *n* (%)	NA	35 (39)	NA
VNS, *n* (%)	8 (18)	3 (3.3)	0.012
MRI abnormalities, *n* (%)	8 (18)	39 (43)	0.001
History cardiac illness, *n* (%)^2^	0	2 (2.2)	NA
Epilepsy etiology, *n*			NA
Structural	0	50	
Genetic	45	13^3^	
Infectious	0	1	
Metabolic	0	0	
Immune	0	1	
Unknown	0	25	
Learning disability, *n* (%)	19 (42)	25 (28)	NA
*SCN1A* variant type, *n* ^4^		Not assessed	NA
Missense	15		
Splice site	2		
Nonsense	12		
Small frameshift deletions	11		
Small frameshift duplications	3		
Gross deletions	2		
Gross duplications	1		
Unknown	2		

ASM, antiseizure medication; IQR, interquartile range; NA, not applicable; VNS, vagal nerve stimulator.

^1^ Reported by cases/caregivers.

^2^ One control with an atrial septum defect type II and one with bigeminy/trigeminy.

^3^ Six controls had a generalized epilepsy syndrome with a presumed genetic etiology (e.g., juvenile myoclonic epilepsy), five a genetic cause of a focal epilepsy/encephalopathy (DEPDC5, GRIN1, SLC6A5, PCDH19, and trisomy 13), one Doose syndrome and one blepharophimosis‐mental retardation syndrome (BMRS).

^4^ One case had a missense variant type and a small frame shift deletion, and two cases had both a small frameshift deletion and insertion.

### Seizures

**Figure 2:**
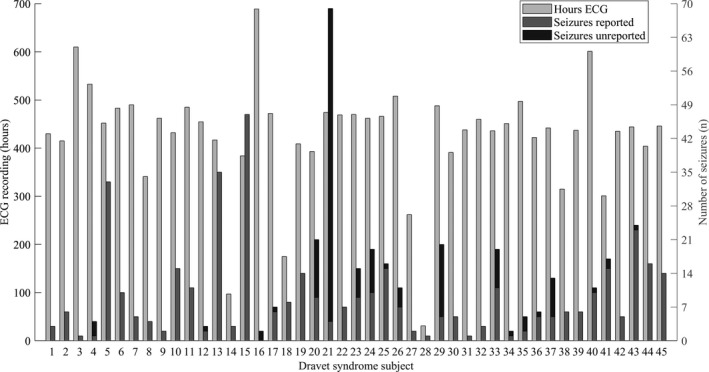
Hours ECG, number of recorded seizures, and proportion unreported seizures for each Dravet syndrome case.

Continuous ECG during a total of 19,174 h was recorded in the 45 cases (mean 426 h per case; Fig. 2). A total of 547 seizures were captured, resulting in a median of seven seizures per person (range 1–69). Seizure types recorded in cases were: 300 CS (55%), 33 tonic (6%), 12 focal seizures with impaired awareness (2.2%), nine focal motor seizures (1.6%), seven hemiclonic (1.3%), one clonic (0.2%), 41 unknown type (7.5%), and 144 unreported seizures (26%; Table 2). Of all 547 seizures, 77 arisen from wakefulness (14%) and 470 from sleep (86%). Most unreported seizures were from sleep (135, 94%). None of the recorded seizures in cases occurred during a period of illness or fever.

In the 90 controls, 169 seizures were recorded with a median of one seizure per person (range 1–8). Seizure types recorded were: 120 CS (71%), 29 tonic seizures (17%), 18 focal seizures with impaired awareness (11%), and two hemiclonic seizures (1.2%). Of the 169 seizures, 67 were from wakefulness (40%) and 102 from sleep (60%).

### Electrocardiographic findings

#### Baseline 12‐lead ECG

In five people with DS, abnormalities were found in the baseline ECG: atrial rhythm (*n* = 2), right axis deviation (*n* = 1), ST‐segment abnormalities in all leads (*n* = 1), and negative T‐waves in V1‐4 (*n* = 1) leading to two referrals for further cardiac evaluation (ST‐segment abnormalities and negative T‐waves). No signs of cardiomyopathy were found in the case with negative T‐waves.

#### Analysis part 1: Peri‐ictal bradyarrhythmias and QTc‐intervals

Asystole was not seen. Bradycardia was more common in seizures of controls (11 seizures in eight subjects, 6.5% of seizures) compared to cases (four seizures in two subjects, 0.7% of seizures; *P* = 0.002; Table 3). In the controls, five of these 11 seizures were CS and six were tonic. In cases, all four were tonic seizures.

**Table 3:** Peri‐ictal electrocardiographic findings in the Dravet syndrome and historical epilepsy control group.

	Dravet syndrome *n* = 45 subjects	Controls *n* = 90 subjects	*P*‐value	95% CI of OR
Seizure types, *n* seizures (%)				
Total number of seizures	547	169	NA	NA
Convulsive	300 (55)	120 (71)	NA	NA
Tonic	33 (6)	29 (17)	NA	NA
Focal impaired awareness	12 (2.2)	18 (11)	NA	NA
Focal motor	9 (1.6)	0	NA	NA
Hemiclonic	7 (1.3)	2 (1.2)	NA	NA
Clonic	1 (0.2)	0	NA	NA
Unknown type reported	41 (7.5)	0	NA	NA
Unreported	144 (6)	0	NA	NA
Peri‐ictal ECG variables, *n* seizures (*n* people; %)				
Bradycardia, *n* seizures (*n* subjects; %)	4 (2; 0.7)	11 (8; 6.5)	0.002	1.2 to 5.3
Prolonged QTc, *n* seizures (*n* subjects; %)	1 (1; 0.2)	1 (1; 0.6)	0.7^1^	−0.99 to 2.8
T1	0	0		
T2	0	1		
T3	1	0		
T4	1	0		
Shortened QTc, *n* seizures (*n* subjects; %)	31 (12; 5.7)	12 (12; 7.1)	0.82^1^	−0.72 to 0.92
T1	17	5		
T2	5	4		
T3	10	3		
T4	5	2		
Ictal QTc‐lengthening, ≥60 ms compared to T1, *n* seizures (*n* subjects; %)	64 (23; 12)	8 (8; 4.7)	0.048^1^	−1.7 to −0.21
T2	53	4		
T3	4	1		
T4	9	3		
Ictal QTc‐shortening, ≥60 ms compared to T1, *n* seizures (*n* subjects; %)	15 (7; 2.7)	13 (11; 7.7)	0.39^1^	−0.26 to 2
T2	12	10		
T3	3	5		
T4	2	4		

CI, confidence interval; OR, odds ratio; T1, time of seizure onset; T2, seizure end; T3, 2 min after seizure end; T4, 5 min after seizure end.

^1^ The Holm–Bonferroni method was used to correct for the multiple comparisons of the QTc‐interval; corrected p‐values and original CIs are shown. Generalized estimating equations were used to correct for within‐subject correlation, seizure onset from sleep or wakefulness and seizure type (convulsive seizure yes or no). QTc changes can occur at multiple time points within seizures.

Peri‐ictal QTc‐lengthening of ≥60 ms was more common in cases (64 seizures in 23 subjects, 12% of seizures) than controls (eight seizures in eight subjects, 4.7% of seizures; *P* = 0.048 (*P* = 0.012*4)). Within the Dravet group, the median age did not differ between those with QTc‐lengthening (*n* = 23; median 14 years, IQR 12–21) and those without (*n* = 22; median 18 years, IQR 14–25; *P* = 0.12). Anti‐seizure medications and the presence of ictal QTc‐lengthening of ≥60 ms or bradycardia, for cases and controls are provided in Table S2. No difference was found in the number of seizures with ictal QTc‐shortening of ≥60 ms between cases (15 seizures in seven subjects, 2.7%) and controls (13 seizures in 11 subjects, 9.5%; *P* = 0.39 (*P* = 0.13*3)). The occurrence of prolonged (cases *n* = 1, 0.2% vs. controls *n* = 1, 0.6%; *P* = 0.7 (*P* = 0.35*2)) and shortened QTc (cases *n* = 31, 5.7% vs. controls *n* = 12, 7.1%; *P* = 0.82 (*P* = 0.82*1)) also did not differ between the groups. Marked prolongation and shortening did not occur in either group.

#### Analysis part 2: Peri‐ictal heart rates, heart rate variability, QRS‐width, and PR‐interval in convulsive seizures

In 41 cases, 300 CS and in 72 controls, 120 CS were analyzed. In cases, 56 CS (19%) were from wakefulness and 244 (81%) from sleep and in the controls 49 CS (42%) from wakefulness and 71 from sleep (59%). Mean age in this selection of the Dravet group was 19 years (±11 years) and 20 were female (49%). In controls, the mean age was 21 years (±9.3 years) and 35 were female (49%).

**Figure 3:**
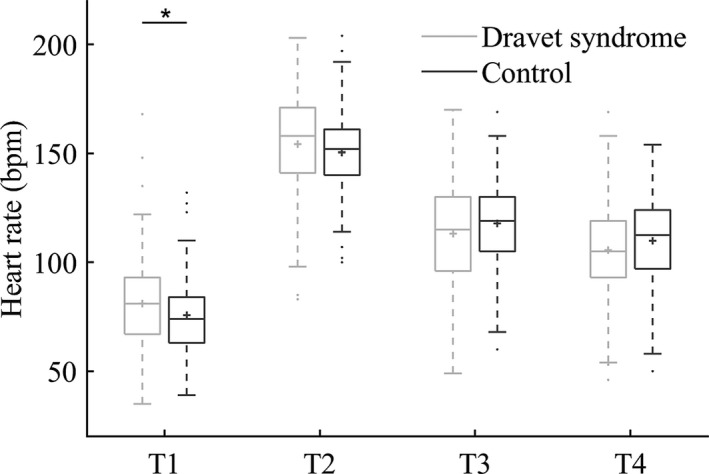
Box plots of peri‐ictal heart rates in convulsive seizures of the Dravet syndrome and historical epilepsy control groups. T1 = time of seizure onset; T2 = seizure end; T3 = two minutes after T2; T4 = 5 min after T2; * significance *P* < 0.05. Median (solid line), mean (plus sign), interquartile interval (box), minimum, maximum (whiskers, 1.5 IQR), and suspected outliers (dots) are shown. Generalized estimating equation linear models were used to compare heart rates between the groups, correcting for within‐subject correlation and seizure onset from sleep or wakefulness.

**Figure 4:**
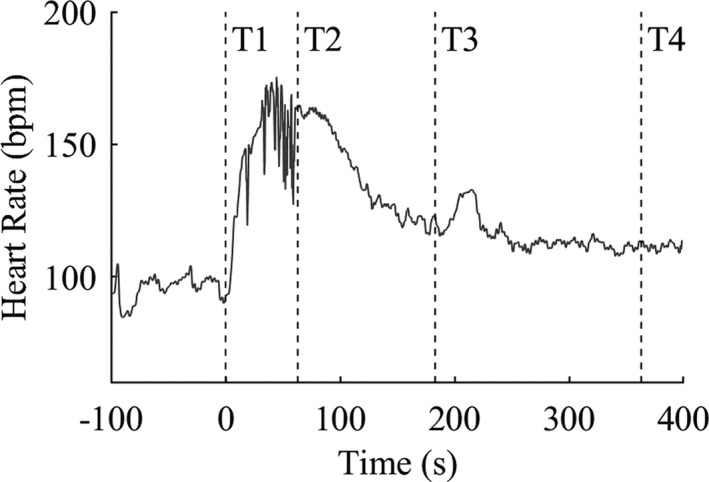
Heart rate curve during a convulsive seizure of a 14‐year‐old girl with Dravet syndrome from 1 min prior to seizure onset to 5 min after seizure end. T1 = time of seizure onset; T2 = seizure end; T3 = 2 min after T2; T4 = 5 min after T2.

At T1, there was a trend of higher mean HR in DS (81 bpm ± 19) compared to controls (76 bpm ± 17, *P* = 0.092; Fig. 3). At the other time points, mean HR did not differ between cases and controls: T2 154 bpm (±23) versus 151 bpm (±20; *P* = 0.75); T3 113 bpm (±24) versus 118 bpm (±22; *P* = 0.92); T4 106 bpm (±22) versus 110 bpm (±21; *P* = 0.39; corrected for within‐person correlation and onset sleep/wakefulness). An example of a continuous HR curve during a CS in a case is shown in Figure 4.

The median resting‐state HRV variable RMSSD was lower in Dravet (37 ms) than in controls (51 ms; *P* = 0.029). Other resting‐state HRV variables were not significantly lower in DS: SDNN was 40 ms in Dravet and 55 ms in controls (*P* = 0.052) and pNN50 was 16 ms in Dravet and 32 ms in controls (*P* = 0.06). The Mean resting‐state HR was higher in cases (RR 740 ms) than in controls (RR 884 ms; *P* < 0.001). Mean HR and HRV variables of the pre‐ and post‐ictal minute did not differ between groups (Table 4).

**Table 4:** Heart rate variability in rest and before and after convulsive seizures in the Dravet syndrome and historical epilepsy control groups.

HRV variables (ms)	Dravet syndrome *n* = 41	Controls *n* = 72	*P*‐value	95% CI
Awake rest				*CI of difference*
Number of people	41	66	NA	NA
RR interval, mean (SD)	740 (140)	884 (175)	<0.001	80 to 208
RMSSD, median (IQR)^1^	37 (20–58)	51 (33–76)	0.029	−15 to 26
SDNN, median (IQR)^1^	40 (23–60)	55 (37–68)	0.052	−10 to 22
pNN50, median (IQR)	16 (1.2–42)	32 (12–52)	0.06	0.14 to 19
Pre‐ictal				*CI of OR*
Number of seizures	285	100	NA	NA
RR interval, mean (SD)	769 (201)	821 (202)	0.18	2 to 168
RMSSD, median (IQR)^1^	46 (21–107)	44 (26–81)	1	−0.1 to 0.09
SDNN, median (IQR)^1^	46 (26–99)	51 (31–73)	1	−0.07 to 0.08
pNN50, median (IQR)^2^	22 (2.4–58)	20 (4.9–51)	0.99	−11 to 11
Postictal				
Number of seizures	288	117	NA	NA
RR interval, mean (SD)	582 (151)	530 (105)	0.34	−98 to 6
RMSSD, median (IQR)^1^	21 (8.8–66)	17 (6.3–52)	0.8	−0.24 to 0.07
SDNN, median (IQR)^1^	29 (15–59)	27 (14–50)	0.68	−0.13 to 0.09
pNN50, median (IQR)^2^	3.2 (0–31)	1.7 (0–16)	0.72	−12 to 5

The Holm–Bonferroni method was used to correct for multiple comparisons within each epoch. Corrected p‐values and original CIs are shown. Resting‐state HRV variables were compared using two‐sided unpaired *t*‐tests or Mann–Whitney U test. Peri‐ictal HRV variables were compared using generalized estimating equation linear models, correcting for within‐person correlation and seizure onset from sleep or wakefulness.

CI, confidence interval; OR, odds ratio; HRV, heart rate variability; IQR, interquartile range; NA, not applicable; pNN50, proportion of pairs of successive RR intervals that differ ≥50 ms; RMSSD, root mean square of successive differences of RR intervals; SDNN, standard deviation of RR intervals.

^1^ Logarithmic transformation was applied to RMSSD and SDNN.

^2^ pNN50 variable was treated as normal distribution in the model as no distribution type fitted original or transformed data.

Peri‐ictal prolonged QRS did not occur in the CS of cases while in controls one mild prolongation was seen (at T1, 2 ms above normal limit). Prolonged PR was also more common in controls (*n* = 3) than in DS (*n* = 1). The case had a mildly prolonged PR interval in one CS at T1 (1 ms above normal limit). The first control had prolonged PR in one CS at T4 (3 ms above normal limit), the second subject in one of six CS at T1 and T4 (by 17 ms and 6 ms), and the third control in one CS at T1 and T4 (by 3 ms and 7 ms).

## Discussion

We prospectively recorded peri‐ictal ECG of 547 seizures in a cohort of 45 people with DS and did not identify actionable major arrhythmias. Peri‐ictal QTc‐lengthening of ≥60 ms was, however, more prevalent in DS compared to controls, occurring in over half of the cases. In line with previous reports, interictal HRV was lower in DS compared to controls.

### Strengths and limitations

The strength of our study is that despite DS being a rare disease, a substantial cohort was recruited. This, combined with the long duration of ECG recordings, resulted in a large overall number of recorded seizures in the home setting. Another strength is that all ECG data were assessed manually for abnormalities. Our Dravet cohort might be an enriched selection, as SUDEP in DS mostly affects young children.^2^ The reported SUDEP cases in DS are, however, likely to be biased toward the young, as improved genetic testing and awareness over the last decades has enabled increased early diagnosis. A younger cohort might have exhibited a higher SUDEP risk, but the high frequency of CS alone places them at high risk for SUDEP.^28^, ^29^ The age of cases with and without QTc‐lengthening did not differ. The age factor therefore does not appear to influence conclusions of this study.

Seizure diaries are known to be unreliable.^30^ Some seizures, and thus potential ictal arrhythmias, within our cohort may therefore have been missed. To overcome this, the complete recordings were meticulously inspected for likely CS, and many unreported seizures were identified. These unreported seizures were not included in the second part of the analysis to ensure only definite CS were involved. Ideally, controls should resemble the Dravet phenotype, with refractory epilepsy and learning disabilities, and be recorded prospectively at home to avoid contrasts in physiological state (e.g., sleep deprivation or drug tapering during clinical stay). Equal recording methods would also enable blinding for analysis, which was not possible due to different data sources. The study burden would, however, be of concern in view of the young age and behavioral problems. The burden would not be outweighed by evidence for increased propensity for arrhythmias, as in DS. The controls here, however, were predominantly people with refractory epilepsy and learning disabilities were present in a considerable proportion – strengthening the power of our study population and supporting the validity of our results.

### Cardiac function in Dravet syndrome

Peri‐ictal QTc‐lengthening of ≥60 ms in DS was up to four times more common than in other epilepsy syndromes.^24^, ^31^, ^32^ The lengthening may result from a less stable cardiac repolarization in DS that increases the propensity for malignant tachyarrhythmias.^33^ Peri‐ictal QTc‐lengthening was mostly brief and often resolved right after seizure end. Peri‐ictal respiratory dysfunction is common in people with DS.^14^ Studies in healthy people showed that hypoxia and hypercapnia can prolong QTc.^34^ Results of studies comparing QTc changes in seizures with and without an SpO^2^ drop of <90% were conflicting: one found more QTc‐shortening and lengthening in the desaturation group,^35^ while the other found no differences.^32^ A recent larger study, using a mixed‐effect model, that, unlike previous studies, included SpO^2^ as a continuous variable and HR and peri‐ictal phase as covariates, found that peri‐ictal QTc changes strongly correlated with SpO^2^.^36^ The QTc‐lengthening found in our cohort may thus be explained by ictal hypoxemia rather than unstable repolarization due to the *SCN1A* mutation. Alternatively, QT changes may be related to contrasts in ASM profiles. With this cohort size and high rates and heterogeneity of ASM polytherapy, we were insufficiently powered to assess the effect of separate ASM types on the study outcomes.

Prolongation of QTc can provoke potentially lethal tachyarrhythmias,^33^ but whether peri‐ictal QTc‐prolongation relates to SUDEP is unclear. Only two SUDEP and four near SUDEP cases in which tachyarrhythmias played a role have been reported.^37^, ^38^, ^39^ A prospective study of out‐of‐hospital cardiac arrests due to ECG‐documented ventricular tachycardia or fibrillation showed a threefold increased risk of these arrhythmias in people with epilepsy compared to the general population.^40^ Most arrhythmias were not seizure‐related and occurred in the context of preexisting or acute heart disease, but some were unexplained and classified as (near) SUDEP.^41^ Sudden cardiac arrest and SUDEP may thus partially overlap.

The lower resting‐state HRV in DS compared to controls confirms previous findings.^15^, ^16^, ^17^, ^18^ Resting‐state HR was, however, higher in DS than controls, which was also seen as a trend in the other studies.^15^, ^16^, ^17^ HR increases are correlated with decreases in HRV.^18^, ^26^ Lower resting‐state HRV in Dravet may thus cohere with a higher resting‐state HR. Decreased HRV has been proposed as a SUDEP biomarker as it may lower the propensity to ictal arrhythmias, but direct evidence linking HRV to SUDEP is still lacking. Postictal bradycardia was unexpectedly more common in controls than in DS. Mouse model studies have reported episodes of bradycardia postically prior to death,^12^, ^13^, ^14^ but also during nonfatal seizures.^14^ Ictal bradycardia only occurred if there was apnea or severely decreased breath amplitude of *Scn1a^R1407X/+^* mice.^14^ The role of cardiac dysfunction in SUDEP in people with DS may thus not be as prominent as previous experimental evidence suggested and most likely occurs in response to respiratory dysfunction. Mice with selective knockout of *SCN1A* in brain interneurons only, and not in cardiac myocytes only, can also experience seizures and die spontaneously.^42^ Single‐cell electrophysiology experiments of human‐ and mice‐derived cardiac myocytes did find increased sodium currents and spontaneous contraction rates in DS compared to control cells.^12^, ^43^ It may seem counterintuitive that reduced sodium channels (due to *SCN1A* haploinsufficiency) leads to these findings. This may, however, reflect compensatory overexpression of other sodium channels (i.e., Nav1.5 encoded by *SCN5A*),^12^, ^43^ which would be in line with the QTc‐lengthening we observed. It is not clear, however, how these electrophysiological studies of mice and single mutated cells translate to human cardiac function. Phenotype variability (i.e., types, locations, and mosaicism) is known to affect developmental outcome and epilepsy severity in DS^3^, ^44^, ^45^ and may also apply to cardiac function.

### Clinical implications

Our analysis did not suggest any major peri‐ictal cardiac arrhythmias which directly explain high SUDEP rates in DS. Postictal QTc‐lengthening in the DS cohort is more likely explained by respiratory dysfunction rather than unstable repolarization due to the *SCN1A* mutation. Prospective data to determine whether QTc lengthening and decreased HRV can predict SUDEP risk in DS is warranted. A 10‐year follow‐up of our cohort and additional analyses in case some succumbed to SUDEP will be performed. An important factor underlying SUDEP risk in DS seems to be epilepsy severity. As CS frequency is the most important risk factor, optimizing seizure control and nocturnal supervision, particularly in view of the substantial number of unreported CS in our cohort, are the most effective preventative measures.^28^, ^46^

## Conflict of Interest

RS reports personal fees from UCB Pharma, EISAI, Cyberonics, Bial, Desitin, and LivaNova, RS is member of the editorial board of Epilepsy and Behavior and Epilepsia Open. JWS reports personal fees from UCB and Zogenix, grants from UCB, Eisai, UCB, GW Pharma. JWS's current position is endowed by the Epilepsy Society, he is a member of the Editorial Board of the Lancet Neurology and receives research support from the Marvin Weil Epilepsy Research Fund. JHC reports grants from GW Pharma, Zogenix, Marinius, Vitaflo, Nutricia, and National Institute for Health Research Biomedical Centre at Great Ormond Street Hospital for Children NHS Foundation trust. SS reports personal fees from UK Epilepsy Society. BG reports personal fees from GW Pharmaceuticals, Zogenix, and OVID/Takeda. RDT reports personal fees from UCB, GSK, Theravance, Novartis and Medtronic and grants from Nuts OHRA Foundation, Medtronic, AC Thomson Foundation and The Netherlands Organisation for Health Research and Development (ZonMW). RDT is a member of the editorial board of Seizure, Epilepsia and Clinical Autonomic Research. The remaining authors have no conflicts of interest relevant to this research.

## Ethical publication statement

We confirm that we have read the Journal’s position on issues involved in ethical publication and affirm that this report is consistent with those guidelines.

## Supporting information


**Table S1**. SCN1A variants of the 45 subjects in the Dravet syndrome group.

**Table S2**. Antiseizure medication types and the presence of peri‐ictal QTc prolongation ≥60 ms and postictal bradycardia in ≥1 of the recorded seizures, of Dravet syndrome cases, and historical epilepsy controls.
