# Supplementary material for: Cardiac arrhythmias in Dravet syndrome: an observational multicenter study
Source: Ann Clin Transl Neurol. 2020 Mar 24;7(4):462–73. doi: 10.1002/acn3.51017 (PMC7187713; doi:10.1002/acn3.51017)
Supplement: Supplementary file 1 — Table S1. SCN1A variants of the 45 subjects in the Dravet syndrome group. [file ACN3-7-462-s001.docx]

**Supplementary Table 1.** *SCN1A* variants of the 45 subjects in the Dravet syndrome group.

| **Subject #** | ***SCN1A* variant** | **Variant type**  1 missense  2 splice site  3 nonsense  4 small frameshift deletions  5 small duplications  6 gross deletions  7 gross duplications | **Parents tested negative** |
| --- | --- | --- | --- |
| 1 | c.1738C>T (p.Arg580Stop) | 3 | Yes |
| 2 | c.812G>A (p.Gly271Asp) | 1 | Yes |
| 3 | c.2837G>A (p.Arg946His) | 1 | Yes |
| 4 | c.4573C>T (p.Arg1525Stop) | 3 | Not tested |
| 5 | c.[4289C>A (+) 4551A>G] (p.[Thr1430Lys (+) Lys1517Lys]) (UV) | 1; and a synonymous variant | Yes |
| 6 | c.3430-?_4002+?dup (dup exon17_exon20) | 7 | Yes |
| 7 | c.4841delT (p.Leu1614ProfsX4) | 4 | Yes |
| 8 | c.3790delA (p.Ile1264fs) | 4 | Yes |
| 9 | c.1510_1514delGAAAA (p.Arg504fs) | 4 | Yes |
| 10 | c.4298:G>A (p. Gly1433Glu) | 1 | Yes |
| 11 | c.5536_5539delAAAC (p.Lys1846fs) | 4 | Yes |
| 12 | c.2343T>A (p.Asn781Lys) | 1 | Yes |
| 13 | c.5734C>T (p.Arg1912Stop) | 3 | Not tested |
| 14 | c.5266T>C (p.Cys1756Arg) | 1 | Yes |
| 15 | c.[2113G>T];[=] (p.[(Glu705*)];[(=)] Chr2(GRCh37):g.[166898865C>A;=]) | 3 | Yes |
| 16 | c.2791C>T (p.Arg931Cys) | 1 | Yes |
| 17 | c.[5536_5539del];[=] (p.[(Lys1846fs)]; [(=)]) | 4 | Yes |
| 18 | c.2584C>T (p.Arg862Stop) | 3 | Yes |
| 19 | c.2814C>T (p.Arg1407Stop) | 3 | Yes |
| 20 | del SCN1A exon 2-23 | 6 | Yes |
| 21 | c.[140delA (+) 2770G>A] (p.[Asn47fs (+) Ala924Thr]) | 4 and 1 | Yes |
| 22 | c.4331C>A (p.Ser1444Tyr) | 1 | Yes |
| 23 | c.3542 del T (p. Phe 1181 fs X 1208) | 4 | Yes |
| 24 | c.[383+6delC];[=] (p.[(?)](VUS);[(=)]) | 2 | Yes |
| 25 | c.5013delT (p.Phe1671fs) | 4 | Yes |
| 26 | c.2836G>A (p.Arg946His) | 1 | Not tested |
| 27 | c.1360C>T (p.Gln454X) | 3 | Yes |
| 28 | c.4757G>A (p.Gly1586Glu) | 1 | Not tested |
| 29 | c.[1285C>T];[=] p.[(Gln429*)];[(=)] (Chr2(GRCh37):g.[166903372G>A];[=]) | 3 | Not tested |
| 30 | c.4633A>G (p.Ile1545Val) (UV) | 1 | Yes |
| 31 | c.4904_4905insT (p.Phe1635fsX1641) | 5 | Yes |
| 32 | c.4219C>T (p.Arg1407Stop) | 3 | Yes |
| 33 | c.1178G>A (p.Arg393His) | 1 | Yes |
| 34 | c.987_988delinsC (p.Leu331fs) | 4 and 5 | No, mother low graded mosaicism |
| 35 | c.2837G>A (p.Arg946His) | 1 | Not tested |
| 36 | c.5674C>T (p.Arg1892Stop) | 3 | Yes |
| 37 | c.3762T>A (p.Tyr1254Stop) | 3 | Not tested |
| 38 | C.383+5C>T | 2 | Yes |
| 39 | 2q24.3-q31.1 | 6 | Yes |
| 40 | c.1837C>T (p.Arg613Stop), Exon 11 | 3 | Not tested |
| 41 | c.1177C>T (p.Arg393Cys), Exon 9 | 1 | Yes |
| 42 | c.5536-5539 delAAAA (pLys 1846fsX.1858) | 4 | Yes |
| 43 | c.3458-3459delAAins CTACTGT | 4 and 5 | Yes |
| 44 | Unknown | Unknown | Yes |
| 45 | Unknown | Unknown | Yes |
