# Supplementary material for: Cardiac arrhythmias in Dravet syndrome: an observational multicenter study
Source: Ann Clin Transl Neurol. 2020 Mar 24;7(4):462–73. doi: 10.1002/acn3.51017 (PMC7187713; doi:10.1002/acn3.51017)
Supplement: Supplementary file 2 — Table S2. Antiseizure medication types and the presence of peri‐ictal QTc prolongation ≥60 ms and postictal bradycardia in ≥1 of the recorded seizures, of Dravet syndrome cases, and historical epilepsy controls. [file ACN3-7-462-s002.docx]

**Supplementary Table 2.** Antiseizure medication types and presence of peri-ictal QTc prolongation ≥60ms and postictal bradycardia in ≥1 of the recorded seizures, of Dravet syndrome cases and historical epilepsy controls.

| **Dravet syndrome (n = 45)** | | | | | | **Controls (n = 90)** | | | | | | |
| --- | --- | --- | --- | --- | --- | --- | --- | --- | --- | --- | --- | --- |
| **Subject** | **Age** | **Medications** | **QTc ↑** | | **Brady** | **Subject** | **Age** | **Medications** | | **QTc ↑** | **Brady** |  |
| 1 | 22 | CLB, TPM, VPA | Yes | |  | 1 | 18 | LCM, OCB | |  |  |  |
| 2 | 15 | - |  | |  | 2 | 22 | CLB, LTG, OCB | |  |  |  |
| 3 | 32 | CLB, VPA |  | |  | 3 | 35 | LTG, VPA | | Yes |  |  |
| 4 | 66 | CLP, VPA |  | |  | 4 | 63 | CBZ, LTG | |  |  |  |
| 5 | 14 | CLB, STI, TPM, VPA | Yes | |  | 5 | 61 | VPA | |  |  |  |
| 6 | 13 | PHN, TPM, VPA |  | |  | 6 | 39 | CBZ, LEV, VPA | |  |  |  |
| 7 | 7 | CLB, LEV, STI, VPA |  | |  | 7 | 34 | CBZ, LEV, LTG | |  |  |  |
| 8 | 20 | CLB, LEV |  | |  | 8 | 33 | VPA | |  |  |  |
| 9 | 18 | LEV, TPM, VPA |  | |  | 9 | 34 | CBZ, LCM | |  |  |  |
| 10 | 17 | CBD, STI, TPM | Yes | |  | 10 | 30 | LTG, LEV, OCB, VPA | |  |  |  |
| 11 | 11 | CBD, CLB, LEV, STI, VPA | Yes | |  | 11 | 30 | LEV | | Yes | Yes |  |
| 12 | 25 | CLB, FLB, VPA |  | |  | 12 | 29 | CLB, LCM | |  |  |  |
| 13 | 13 | CLB, TPM, VPA | Yes | |  | 13 | 27 | CLB, VPA | | Yes | Yes |  |
| 14 | 7 | CBD, CLB, LEV, ZNS | Yes | |  | 14 | 25 | - | |  | Yes |  |
| 15 | 12 | CLB, VPA | Yes | |  | 15 | 25 | CBZ, LEV, LTG | |  |  |  |
| 16 | 23 | CLB, VPA |  | |  | 16 | 30 | CBZ, CLB, VPA | |  |  |  |
| 17 | 39 | CBZ, PRI, TPM, VPA | Yes | |  | 17 | 27 | CLB, LTG | |  |  |  |
| 18 | 8 | CLB, STI, VPA | Yes | | Yes | 18 | 25 | LTG | |  |  |  |
| 19 | 21 | CLB, STI, VPA | Yes | |  | 19 | 23 | CBZ, LTG | |  |  |  |
| 20 | 25 | CLB, OCB, TPM |  | |  | 20 | 23 | LCM | |  |  |  |
| 21 | 20 | VPA, ZNS | Yes | |  | 21 | 27 | LEV, OCB | |  |  |  |
| 22 | 30 | CLB, LEV, TPM, VPA |  | | Yes | 22 | 22 | CBZ,VPA | |  |  |  |
| 23 | 15 | CBD, LEV, TPM, VPA | Yes | |  | 23 | 22 | CLB, LEV, TPM | |  |  |  |
| 24 | 23 | CBZ, LTG, VPA | Yes | |  | 24* | 22 | CLB, LTG, VPA | |  |  |  |
| 25 | 29 | LTG, TPM, VPA | Yes | |  | 25 | 23 | OXZ | | Yes |  |  |
| 26 | 14 | CBD, LEV, STI, VPA | Yes | |  | 26 | 23 | CBZ, VPA | |  |  |  |
| 27 | 16 | CLB, LTG, STI, TPM |  | |  | 27 | 22 | LEV | |  |  |  |
| 28 | 32 | OCB, VPA |  | |  | 28 | 21 | CLB, LEV, VPA | |  |  |  |
| 29 | 22 | VPA | Yes | |  | 29 | 22 | LEV | |  | Yes |  |
| 30 | 12 | CLB, STI, VPA | Yes | |  | 30 | 20 | CBZ, CLP, VPA | | Yes | Yes |  |
| 31 | 14 | CLB, STI, VPA |  | |  | 31 | 20 | LEV, LTG | |  |  |  |
| 32 | 10 | STI, VPA |  | |  | 32* | 21 | CBZ, LCM | |  |  |  |
| 33 | 13 | CLB, STI, VPA |  | |  | 33 | 22 | LTG | |  |  |  |
| 34 | 18 | CLB, PGB, STI, VPA |  | |  | 34 | 21 | LEV | |  |  |  |
| 35 | 10 | CLB, LEV, STI, VPA | Yes | |  | 35 | 20 | LTG | |  |  |  |
| 36 | 16 | CLB, TPM, VPA | Yes | |  | 36* | 23 | LTG, OCB, VPA | |  |  |  |
| 37 | 21 | CLB, STI, TPM, VPA | Yes | |  | 37 | 21 | VPA | |  |  |  |
| 38 | 25 | BRI, CLB, PHB, VPA |  | |  | 38* | 21 | OCB, STI, ZNS | |  |  |  |
| 39 | 6 | STI, VPA | Yes | |  | 39 | 21 | LZP, VPA | | Yes |  |  |
| 40 | 12 | VPA | Yes | |  | 40* | 19 | CLB, LTG, TPM | |  |  |  |
| 41 | 26 | CLB, STI |  | |  | 41* | 19 | CBZ, CLB, LTG | |  |  |  |
| 42 | 11 | CLB, STI |  | |  | 42 | 19 | GBP, OCB, ZNS | |  |  |  |
| 43 | 10 | CBD, CLB, STI |  | |  | 43 | 18 | CBZ, CLB, VPA | |  |  |  |
| 44 | 14 | - |  | |  | 44* | 19 | CLB, OCB | |  |  |  |
| 45 | 15 | CLB, STI, VPA |  | |  | 45 | 19 | CBZ, SLT | | Yes |  |  |
|  |  |  |  | |  | 46 | 25 | OCB | |  |  |  |
|  |  |  |  | |  | 47 | 23 | CBZ, LTG | |  | Yes |  |
|  |  |  |  | |  | 48 | 19 | CBZ | |  |  |  |
|  |  |  |  | |  | 49* | 16 | - | |  |  |  |
|  |  |  |  | |  | 50* | 15 | OCB, VPA | |  |  |  |
|  |  |  |  | |  | 51 | 13 | CBZ, LEV | |  |  |  |
|  |  |  |  | |  | 52 | 14 | OCB, VPA | |  |  |  |
|  |  |  |  | |  | 53 | 15 | LTG | |  |  |  |
|  |  |  |  | |  | 54 | 14 | LCM, LEV, GBP, VPA | |  |  |  |
|  |  |  |  | |  | 55* | 15 | CBZ, LCM, LEV | |  |  |  |
|  |  |  |  | |  | 56* | 17 | ETH, LTG, OCB | |  |  |  |
|  |  |  |  | |  | 57 | 16 | LEV | |  |  |  |
|  |  |  |  | |  | 58* | 12 | LTG, TPM | |  |  |  |
|  |  |  |  | |  | 59* | 17 | CBZ | |  |  |  |
|  |  |  |  | |  | 60 | 18 | CBZ, CLB | |  |  |  |
|  |  |  |  | |  | 61 | 14 | - | |  |  |  |
|  |  |  |  | |  | 62 | 15 | LEV | |  |  |  |
|  |  |  |  | |  | 63 | 13 | LTG | |  |  |  |
|  |  |  |  | |  | 64* | 11 | LTG, VPA, ZNS | |  |  |  |
|  |  |  |  | |  | 65* | 12 | CLP, FLB, LTG, VPA | |  |  |  |
|  |  |  |  | |  | 66* | 11 | CBZ | |  |  |  |
|  |  |  |  | |  | 67 | 11 | LTG, OCB | |  |  |  |
|  |  |  |  | |  | 68* | 15 | CBZ, LTG | |  |  |  |
|  |  |  |  | |  | 69 | 17 | OCB, ZNS | |  |  |  |
|  |  |  |  | |  | 70 | 16 | CLB, LCM, OCB, SLT | |  |  |  |
|  |  |  |  | |  | 71 | 21 | LEV | |  |  |  |
|  |  |  |  | |  | 72* | 10 | - | |  | Yes |  |
|  |  |  |  | |  | 73 | 9 | VPA | |  |  |  |
|  |  |  |  | |  | 74* | 9 | OCB, VPA | |  |  |  |
|  |  |  |  | |  | 75* | 9 | LEV, VPA | |  |  |  |
|  |  |  |  | |  | 76 | 17 | OCB, LTG | |  |  |  |
|  |  |  |  | |  | 77 | 18 | OCB | |  |  |  |
|  |  |  |  | |  | 78 | 10 | OCB | |  |  |  |
|  |  |  |  | |  | 79 | 7 | CLB, LTG | |  |  |  |
|  |  |  |  | |  | 80 | 12 | - | |  |  |  |
|  |  |  |  | |  | 81 | 14 | - | |  |  |  |
|  |  |  |  | |  | 82* | 12 | LTG, VPA | |  |  |  |
|  |  |  |  | |  | 83 | 6 | CLB, LEV, VPA | |  |  |  |
|  |  |  |  | |  | 84* | 12 | LEV, VPA | | Yes |  |  |
|  |  |  |  | |  | 85* | 9 | CLB, OCB | |  |  |  |
|  |  |  |  | |  | 86 | 8 | CBZ, CLB | |  | Yes |  |
|  |  |  |  | |  | 87 | 14 | OCB | |  |  |  |
|  |  |  |  | |  | 88 | 8 | LEV | |  |  |  |
|  |  |  |  | |  | 89 | 18 | CLB, LEV | |  |  |  |
|  |  |  |  | |  | 90* | 16 | CBZ, LEV | |  |  |  |
|  | | | | | | | | | | | | |
| **AED, n (%)** | | | | **Dravet syndrome (n = 45)** | | | | | **Controls (n = 90)** | | | |
| BRI | | | | 1 (2) | | | | | 0 (0) | | | |
| CBD | | | | 6 (13) | | | | | 0 (0) | | | |
| CBZ | | | | 2 (4) | | | | | 24 (27) | | | |
| CLB | | | | 28 (62) | | | | | 19 (21) | | | |
| CLP | | | | 1 (2) | | | | | 2 (2) | | | |
| ETH | | | | 0 (0) | | | | | 1 (1) | | | |
| FLB | | | | 1 (2) | | | | | 1 (1) | | | |
| GBP | | | | 0 (0) | | | | | 2 (2) | | | |
| PHB | | | | 1 (2) | | | | | 0 (0) | | | |
| PHN | | | | 1 (2) | | | | | 0 (0) | | | |
| LCM | | | | 0 (0) | | | | | 8 (9) | | | |
| LEV | | | | 9 (20) | | | | | 24 (27) | | | |
| LTG | | | | 3 (7) | | | | | 28 (31) | | | |
| LZP | | | | 0 (0) | | | | | 1 (1) | | | |
| OCB | | | | 2 (4) | | | | | 21 (23) | | | |
| OXZ | | | | 0 (0) | | | | | 1 (1) | | | |
| PGB | | | | 1 (2) | | | | | 0 (0) | | | |
| PRI | | | | 1 (2) | | | | | 0 (0) | | | |
| SLT | | | | 0 (0) | | | | | 2 (2) | | | |
| STI | | | | 19 (42) | | | | | 1 (1) | | | |
| TPM | | | | 14 (31) | | | | | 3 (3) | | | |
| VPA | | | | 35 (78) | | | | | 27 (30) | | | |
| ZNS | | | | 2 (4) | | | | | 4 (4) | | | |

A “yes” indicates QTc-lengthening or bradycardia is present in one or more seizures of this subject. * Epilepsy control has a learning disability. Brady = bradycardia; QTc ↑ = peri-ictal QTc prolongation of ≥60ms; BRI = brivaracetam; CBD = cannabidiol; CBZ = carbamazepine; CLB = clobazam; CLP = clonazepam; ETH = ethosuxamide; FLB = felbamate; GBP = gabapentin; LCM = lacosamide; LEV = levetiracetam; LTG = lamotrigine; LZP = lorazepam; OCB = oxcarbazepine; PGB = pregabalin; PHB = phenobarbital; PHN = phenytoin; PRI = primidone; SLT = sulthiame; STI = stiripentol; TPM = topiramate; VPA = valproic acid; ZNS = zonisamide.
